# Excellent Room-Temperature Thermoelectricity of 2D GeP_3_: Mexican-Hat-Shaped Band Dispersion and Ultralow Lattice Thermal Conductivity

**DOI:** 10.3390/molecules26216376

**Published:** 2021-10-21

**Authors:** Cong Wang, Zhiyuan Xu, Ke Xu, Guoying Gao

**Affiliations:** 1 School of Physics, Huazhong University of Science and Technology, Wuhan 430074, China; phywangc@hbuas.edu.cn (C.W.); xzy6517@163.com (Z.X.); 2 Hubei Key Laboratory of Low Dimensional Optoelectronic Materials and Devices, Hubei University of Arts and Science, Xiangyang 441053, China; xuke@hbuas.edu.cn

**Keywords:** thermoelectricity, power factor, lattice thermal conductivity, GeP_3_ monolayer, first-principles, Boltzmann transport

## Abstract

Although some atomically thin 2D semiconductors have been found to possess good thermoelectric performance due to the quantum confinement effect, most of their behaviors occur at a higher temperature. Searching for promising thermoelectric materials at room temperature is meaningful and challenging. Inspired by the finding of moderate band gap and high carrier mobility in monolayer GeP_3,_ we investigated the thermoelectric properties by using semi-classical Boltzmann transport theory and first-principles calculations. The results show that the room-temperature lattice thermal conductivity of monolayer GeP_3_ is only 0.43 Wm^−1^K^−1^ because of the low group velocity and the strong anharmonic phonon scattering resulting from the disordered phonon vibrations with out-of-plane and in-plane directions. Simultaneously, the Mexican-hat-shaped dispersion and the orbital degeneracy of the valence bands result in a large *p*-type power factor. Combining this superior power factor with the ultralow lattice thermal conductivity, a high *p*-type thermoelectric figure of merit of 3.33 is achieved with a moderate carrier concentration at 300 K. The present work highlights the potential applications of 2D GeP_3_ as an excellent room-temperature thermoelectric material.

## 1. Introduction

Thermoelectric (TE) materials, which could convert thermal energy into electric energy, have become more and more important due to their potential in resolving the global warming and the energy dilemma [1,2]. The efficiency of TE conversation is quantified by the TE figure of merit *ZT* = *S*^2^*σT*/*κ*. Here, *T* is the temperature, *σ* is the electrical conductivity, *S* is the Seebeck coefficient, and *κ* is the sum of electronic and phonon (lattice) thermal conductivity. These TE coefficients are connected by the carrier concentration and will lead to a maximum *ZT* value at a proper carrier concentration. In the past few years, numerous efforts have suggested that the TE performance of low-dimensional systems could be enhanced compared to their bulk counterparts because of the quantum confinement effect [3,4,5].

Graphene-like atomically thin 2D materials, such as germanene, black phosphorene, arsenene, silicene, and transition-metal dichalcogenides monolayers have attracted intense interest in thermoelectrics recently [6,7,8,9,10,11]. Some of them showed excellent TE performance at a moderate or higher temperature. As we all know, TE materials with higher *ZT* values at room temperature will make them commercially viable for the cooling and power generation field [12]. However, searching for room-temperature 2D high-performance TE materials is still challenging, because the relatively high lattice thermal conductivity at room temperature hinders the increase of the *ZT* value. For example, the optimized *ZT* values at 300 K are only 0.01 for monolayer graphene, 0.16 for monolayer graphyne, 0.14 for monolayer WSe_2_ [7,8]. Although the *ZT* value can reach 1.2 at 1200 K for *p*-type CdPSe_3_, it is only 0.5 at room temperature [9]. Therefore, it is necessary to search for promising room-temperature TE materials.

Latterly, a new type of 2D monolayers MP_3_ (M = Ga, In, Ge, Sn) received growing attention in view of their novel chemical and physical properties [13,14,15,16,17,18,19,20,21]. Their structures are associated with arsenic, which can be regarded as one *M* atom replacing every fourth atom in the arsenic layer and the rest replaced by phosphorus (P) atoms. Interestingly, when the thickness is equal to or greater than three layers, both SnP_3_ and GeP_3_ will become metals due to the correlation between lone-pair electrons of interlayer Ge(Sn) and P atoms [13,14]. Importantly, all the MP_3_ monolayers are predicted to be 2D semiconductors with high carrier mobilities and moderate energy gaps [13,14,15,16], and the biaxial compressive strain can lead to the transition of indirect-direct band gaps for both GeP_3_ and SnP_3_ [13,14,15]. In addition, both GeP_3_ and SnP_3_ monolayers have been predicted as promising candidates for future applications in Li-ion batteries and electrocatalysis [17,18]. Even so, there have been little research on the TE properties for 2D MP_3_ [19,20,21]. We would like to explore and understand the electron and phonon transport properties of 2D GeP_3_ belonging to an MP_3_ family.

In this work, we demonstrate from the first-principles calculations that the GeP_3_ monolayer obtains a superior *p*-type *S*^2^*σ* due to the Mexican-hat-shaped dispersion and the orbital degeneracy for the valence bands. Meanwhile, an ultralow lattice thermal conductivity is obtained and understood from the phonon spectrum, phonon group velocity, Grüneisen parameter, vibration modes of lattice, and three-phonon scattering phase space. The superior power factor and the ultralow lattice conductivity lead to a high *p*-type *ZT* value of 3.33 at 300 K, which indicates the potential of monolayer GeP_3_ as a room-temperature TE material.

## 2. Computational Methods

Our calculations include three parts. Firstly, the structural optimization of the GeP_3_ monolayer is achieved by the VASP code [22]. The generalized gradient approximation (GGA) in the Perdew–Burke–Ernzerhof (PBE) type is adopted for the exchange-correlation functional [23]. A 550 eV is set for the cutoff energy, and a 15 × 15 × 1 q-grid is chosen in the Brillouin zone. We use the hybrid functional (HSE06) [24] to obtain the band structure and the electronic transport properties because the GGA usually underestimates the band gap.

Secondly, the electronic transport properties are computed by the Boltzmann transport theory within the relaxation time approximation in the BoltzTraP code [25]. The *κ_e_* is conducted by the Wiedemann-Franz law *κ_e_* = *LσT*, here, *L* = 2.44 × 10^−8^ WΩK^−2^ represents the Lorentz number [26]. The carrier relaxation time *τ* is estimated by the deformation potential theory, which has been extensively applied for 2D systems [6,27,28]. The calculated electron (hole) relaxation time at room temperature is 1.9 (6.2) × 10^−13^ s, which is close to the results obtained by Zeng et al. [29].

Thirdly, the thermal transport properties are obtained via solving the phonon Boltzmann transport equation with ShengBTE [30]. A 4 × 4 × 1 supercell with 7 × 7 × 1 *k*-mesh is chosen to acquire the second-order and third-order interatomic force constants (IFCs). The cutoff of interaction is up to the sixth nearest neighbors for the anharmonic IFCs. The interlayer separation of bulk is used as the thickness of the monolayer to calculate the lattice thermal conductivity, which has been used in other 2D monolayers [31,32]. It is worth noting that the present computational approaches of transport properties have been reviewed recently by Kozinsky and Singh [33].

## 3. Results and Discussion

**Figure 1:**
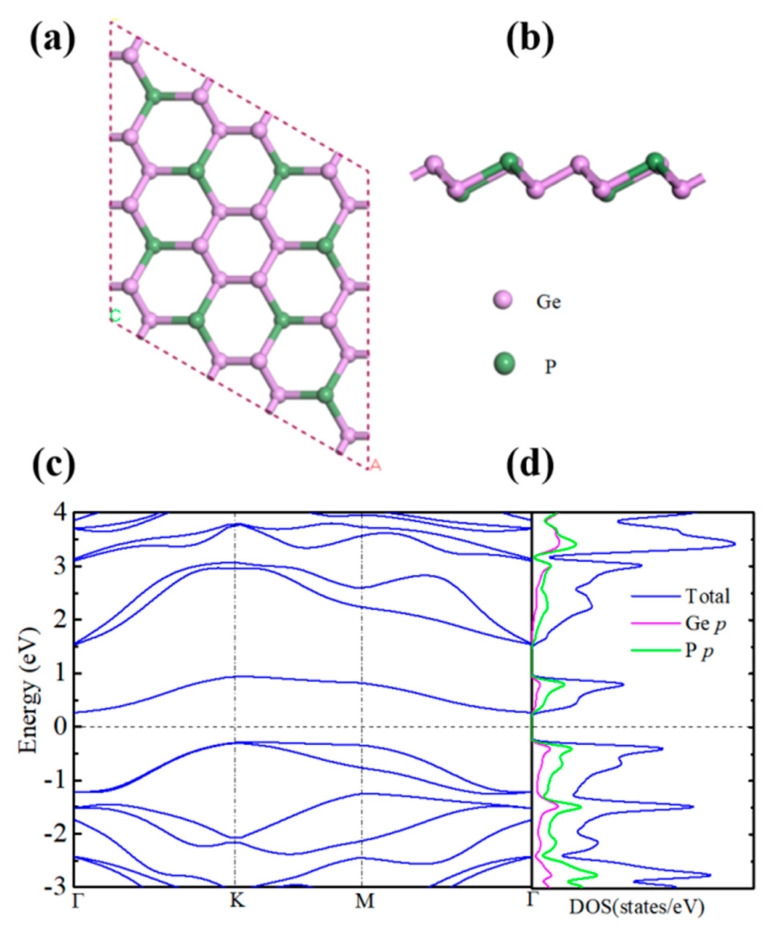
The top (**a**) and side (**b**) views of the crystal structure and the band structure (**c**) and atomic orbit-resolved density of states (**d**) for monolayer GeP_3_. The pink and green balls represent the Ge and P atoms, respectively.

The crystal structure of the GeP_3_ monolayer is plotted in Figure 1a,b. The optimized lattice constant *a = b* = 6.96 Å is closed to the earlier theoretical values [13,17,18]. We first calculate the electronic band structure and the density of states (DOS) within HSE06. It is distinctly seen in Figure 1c that the GeP_3_ monolayer is an indirect band gap (0.53 eV) semiconductor, which is consistent with the value of 0.55 eV reported by Jing et al [13]. The calculated total and atomic orbit-resolved DOS (Figure 1d) reveal that both conduction bands and valence bands derive from the hybridized P and Ge *p* orbitals. The uppermost valence bands emerge so-called “Mexican-hat-shaped” dispersion, which can greatly enhance the TE properties [34,35]. As can be seen from Figure 1c, the “Mexican-hat-shaped” dispersion on the top of valence bands show heavy and light bands simultaneously. The heavy bands along the *M*-*K* direction are very flat, which will lead to a large DOS effective mass and thus, a large Seebeck coefficient. Meanwhile, the light bands with distinct dispersion along *M*-*Γ* and *K*-*Γ* directions will result in high electrical conductivity. Moreover, the two separated bands are degenerate at the *K* point on the top of the valence bands. Such degenerate band structure feature is considered to take great effect on electronic transport and will give rise to a high density-of-state effective mass and in turn a large Seebeck coefficient [36,37].

**Figure 2:**
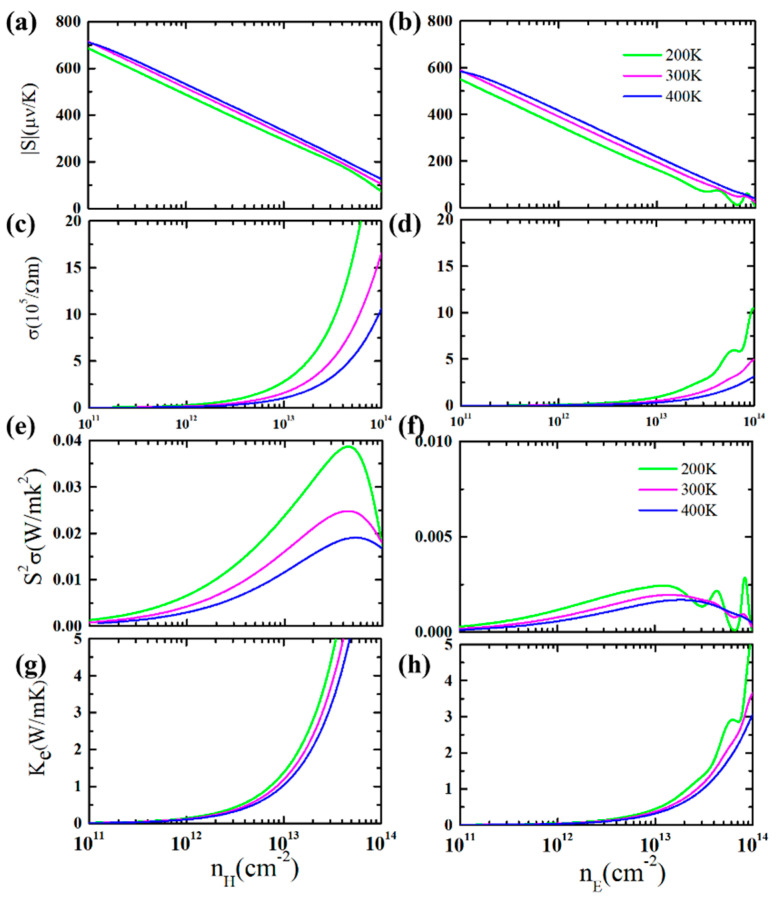
The absolute value of Seebeck coefficient (**a**,**b**), the electrical conductivity (**c**,**d**), the power factor (**e**,**f**), and the electronic thermal conductivity (**g**,**h**) with the change of carrier concentration and temperature for monolayer GeP_3_. The left (right) part represents the *p*-type (*n*-type) doping.

On the basis of the obtained band structure of the GeP_3_ monolayer, we further computed the electronic transport properties including the *S*, *σ*, *PF*, and *κ_e_*. We simulate the doping by the rigid band approximation. The obtained *σ* and *S* with the change of carrier concentration *n* at three different temperatures are shown in Figure 2a–d. As can be seen, the Seebeck coefficient of *p*-type doping is larger than that of *n*-type doping due to the flat valence bands along the *M*-*K* direction and the band degeneracy at the *K* point. With assured carrier concentration, the Seebeck coefficient increases with the increase of temperature. Besides, the *p*-type doping *σ* is much larger than that of *n*-type doping because of the strong dispersion along the *M*-*Γ* and *K*-*Γ* directions. It is natural the maximum power factor can be achieved in *p*-type because a larger Seebeck coefficient and higher electrical conductivity are obtained in *p*-type simultaneously. By comparing the *S* and *σ* values of the *p*-type and *n*-type for monolayer GeP_3_, it is found that the “Mexican-hat-shaped” dispersion for valence band structure can not only significantly increase the *S* by the flat band, but also avoid the poor performance of the σ. Figure 2e, f shows the power factor *S^2^σ*. It can be seen that the *p*-type *S*^2^*σ* is much larger than that of the *n*-type one, which implies that greater TE performance could be achieved by the *p*-type doping. Typically, the obtained room-temperature *p*-type *S*^2^*σ* is about 0.0245 Wm^−1^K^−2^ for monolayer GeP_3_, which is even larger than the well-known TE material Bi_2_Te_3_ (0.003 Wm^−1^K^−2^) [38]. In addition, the plotted electronic thermal conductivity *κ_e_* (Figure 2g,h) indicates that the changes of *σ* and *κ_e_* with temperature and carrier concentration are uniform due to their correlation according to the Wiedemann–Franz law [26].

**Figure 3:**
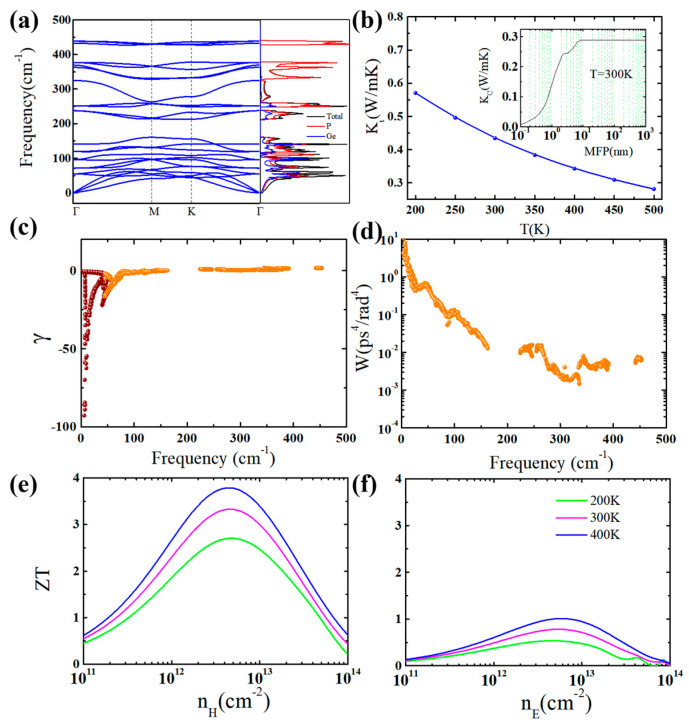
The phonon dispersion (**a**), the temperature-dependent lattice thermal conductivity (**b**), the frequency-dependent Grüneisen parameter γ (**c**) and three-phonon scattering phase space *W* (**d**) at 300 K, and the *p*-type (**e**) and *n*-type (**f**) figure of merit *ZT* with the change of carrier concentration and temperature for monolayer GeP_3_. The inset in (**b**) shows the cumulative lattice thermal conductivity as a function of phonon mean-free-path (MFP) at 300 K.

After attaining the electronic transport properties of the GeP_3_ monolayer, we now examine the phonon transport property. The obtained phonon band structure and phonon density of states of GeP_3_ monolayer are presented in Figure 3a. No imaginary phonon frequency appears which implies the stability of monolayer GeP_3_. The lowest three phonon modes at the Γ point are acoustic phonon branches, i.e., the in-plane transverse acoustic branch (TA), the *z*-direction acoustic mode (ZA), and the in-plane longitudinal acoustic branch (LA). The maximum frequency of acoustic branches is only 1.81 THz, which is comparable or lower than those of admirable TE materials, such as PbTe and SnSe [39], indicating a smaller phonon group velocity in monolayer GeP_3_. In addition, the flat phonon dispersion characteristics of acoustic branches closed to the Γ point is also a key to obtaining a small phonon group velocity and a low lattice thermal conductivity. As expected, the group velocities that could be computed by ∂ω/∂k are 1.70, 1.27 and 1.63 kms^−1^ for the LA, ZA and TA branches at the Γ point, respectively, which are smaller than those of PbTe, SnSe [39], and SnTe [10]. Meanwhile, the low-frequency optical branches are overlapped and mixed with the acoustic modes, resulting in strong interactions and large three-phonon processes, which will have a great influence on the *κ_l_* of monolayer GeP_3_. The calculated total and partial phonon DOS show that the low-frequency optical branches and the acoustic modes are derived from the vibrations of the *Ge* and *P* atom while the *P* atom forms the high-frequency optical modes. So, the thermal carry through the *Ge*–*P* bond is the major heat transfer means in the GeP_3_ monolayer. Due to the flat phonon dispersion closed to the Γ point for acoustic branches, the PhDOS is truly sharp, and consequently, a low *κ_l_* could be anticipated.

**Figure 4:**
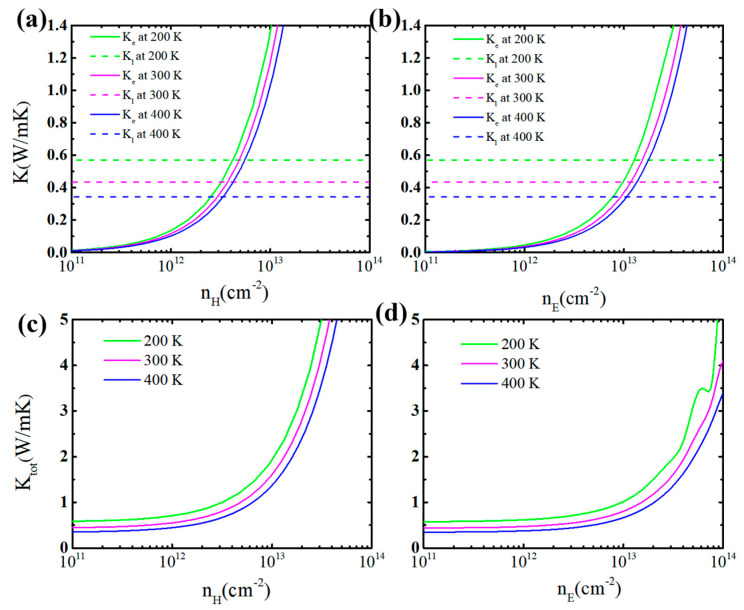
The electron (*κ_e_*) and phonon (*κ_l_*) thermal conductivity (**a**,**b**) as well as the total (*κ_tot_*) thermal conductivity (**c**,**d**) with the change of carrier concentration and temperature for monolayer GeP_3_. The left (right) part represents the *p*-type (*n*-type) doping.

The *κ_l_* which is computed by solving the phonon Boltzmann transport equation performed in ShengBTE [30] can be expressed as

(1) $$\kappa_{L,\alpha \beta }=\sum_{qs}C_{V}\left(qs\right)\upsilon_{g}^{\alpha }\left(qs\right)\upsilon_{g}^{\beta }\left(qs\right)\tau_{qs}$$

where *q*, *s*, *C_V_*, *v_g_*, and *τ* represent the wave vector, the dispersion branch, the phonon mode volumetric specific heat, the group velocity, and the phonon lifetime, respectively. The calculated *κ_ι_* for monolayer GeP_3_ as a function of temperature *T* is plotted in Figure 3b. It can be seen that the *κ_l_* for the GeP_3_ monolayer is fairly low, especially at a high temperature. Typically, the *κ_l_* for the GeP_3_ monolayer is only 0.43 Wm^−1^K^−1^ at 300 K, which is much lower than those of good TE materials of SnSe (0.62 Wm^−1^K^−1^) and PbTe (2.30 Wm^−1^K^−1^) [39], as well as 2D CdPSe_3_ (1.25 Wm^−1^K^−1^) [9]. Such low *κ_l_* means that monolayer GeP_3_ could be a favorable room-temperature TE material. Note that, in our calculations based on the phonon Boltzmann transport equation (ShengBTE code) [30], the phonon-phonon, the isotope and the boundary scatterings are included, but the carrier-phonon scattering is not considered. So, the lattice thermal conductivity *κ_l_* is independent of the carrier concentration *n*. In Figure 4, we present a comparison of the calculated *κ_l_* and *κ_e_*. One can see that the characteristics of (*κ_e_* + *κ_l_*)~*n* is similar to that of *κ_e_*~*n*. The *κ_l_* is higher than the *κ_e_* at the low *n*, but it is opposite at the high *n*.

To get more perception for the phonon thermal transport properties, we calculate the three phonon scattering phase space *W* and Grüneisen parameter *γ* for monolayer GeP_3_, as plotted in Figure 3c,d. The *γ* could qualitatively characterize the anharmonic phonon scattering, and the *W* could give the comprehension of the number of available channels for anharmonic phonon scattering. Usually, larger |*γ*| means strong anharmonic phonon scattering and thus leads to a low *κ*_l_. The calculated average value of the Grüneisen parameter |*γ*| for acoustic branches is 9.6, which is high and indicates the strong anharmonicity and thus a low *κ_l_* can be obtained for monolayer GeP_3_. In addition, it is found that the large Grüneisen parameter and three phonon phase space mainly exist in the low-frequency region, especially for the acoustic branches, which reveals that the acoustic branches contribute most to the total lattice thermal conductivity.

**Figure 5:**
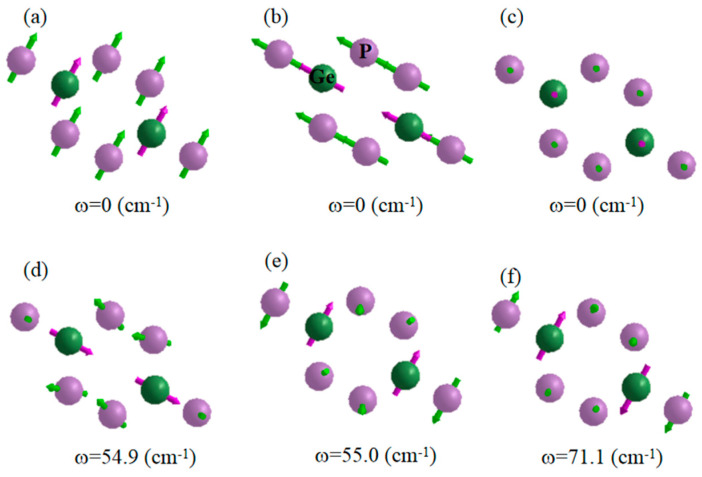
The typical vibrational modes of the acoustic phonon branches (LA (**a**), TA (**b**) and ZA (**c**)) as well as three low-frequency optical phonon branches (**d**–**f**) for monolayer GeP_3_ at the Γ point. The arrows denote the atomic displacement directions at 300 K.

Besides, we study the phonon vibration modes in terms of the computed 2nd IFCs. We show in Figure 5 some typical vibration modes of monolayer GeP_3_ at Γ point. The phonon modes presented in Figure 5a–c are the vibration of three acoustic modes (LA, TA and ZA, respectively). The results show that the vibration directions of these branches are firmly along the out-of-plane (ZA) or the in-plane (LA and TA). Figure 5d is the vibration type of the lowest optical mode. In this mode, the Ge atoms are fixed, some P atoms vibrate out-of-plane, while other P atoms move in-plane in different directions. Similar phonon vibration modes could also be observed in higher-frequency optical branches (Figure 5e,f). These optical modes are effortlessly thermally activated at 300 K and seriously hinder thermal transport as a result of phonon-phonon scattering, and thus, a low *κ_l_* is gained for monolayer GeP_3_.

Nanostructure is one of the important ways to enhance *ZT* value by hindering phonon transport [3,4,5]. The phonon boundary scattering would greatly depress the *κ_ι_* as long as the length of the nanostructure is as short as the phonon mean free path (MFP). Therefore, the discussion on phonon MFP is necessary to study the size effect and also significant to design nano TE devices. The calculated cumulative lattice thermal conductivity (*κ_C_*) in regard to MFP at 300 K for monolayer GeP_3_ is shown in the inset of Figure 3b. The calculated *κ_C_* keeps increasing as the increase of MFP till the thermodynamic limit is reached. The critical value of MFP (*l*_0_) to the maximum lattice thermal conductivity at 300 K is only 8.2 nm. In general, nanostructures with *l*_0_ short than 10 nm is favorable for their TE performance. By comparing with other TE materials, monolayer GeP_3_ shows a fairly low *l*_0_ value. Thus, the intrinsically short MFP for monolayer GeP_3_ hinders the potential for further decrease of *κ_ι_*. In other words, the effect of size on *κ_ι_* for monolayer GeP_3_ is not so significant.

Finally, according to the above calculated thermal transport coefficients, we computed the *ZT* value with the change of temperature and carrier concentration. Note that, the present *ZT* is an estimation based on the relaxation time approximation, because the carrier relaxation time is complex, and it is approximated as a constant in the present calculations by the BoltzTraP code [25] based on the Boltzmann transport theory. As shown in Figure 3e,f, the optimal *ZT* value increases with the increasing temperature. The *p*-type has high *ZT* value than the *n*-type because of the large power factor of *p*-type than *n*-type. For the *p*-type doping at 300 K, the optimal *ZT* value for monolayer GeP_3_ is as large as 3.33 with a large Seebeck coefficient of 400 µV/K at the hole concentration of 5.07 × 10^12^ cm^−2^, while the value is only 0.78 for the *n*-type doping. Therefore, monolayer GeP_3_ is an excellent *p*-type room-temperature TE material due to the high-power factor and the low thermal conductivity. The optimal *p*-type *ZT* value of 3.33 at 300 K is higher than most of 2D semiconductors, such as *p*-type CdPSe_3_ (0.5 at 300 K) [9], *p*-type InP_3_ (2.06 along the armchair direction at 300 K) [19], and *n*-type α-In_2_Se_3_ (2.18 at 300 K) [40], etc.

## 4. Conclusions

In summary, thanks to the recent finding of monolayer GeP_3_ with high carrier mobility and a moderate energy gap, we perform the first principles to explore the electron and phonon transport properties for monolayer GeP_3_. The Mexican-hat-shaped dispersion and the band degeneracy of valence bands result in a high *p*-type power factor. The low frequency of acoustic branches, low phonon group velocity, large Grüneisen parameter and large three-phonon scattering phase space result in an ultralow lattice thermal conductivity of 0.43 Wm^−1^K^−1^ at 300 K. Therefore, a high *p*-type *ZT* value of 3.33 is achieved at 300 K, which is higher than most of the 2D semiconductors, making monolayer GeP_3_ an excellent room TE material.

## Data Availability

Not applicable.

## Sample Availability

Samples of the compounds are not available from the authors.
